# Increased susceptibility to intensive care unit-acquired pneumonia in severe COVID-19 patients: a multicentre retrospective cohort study

**DOI:** 10.1186/s13613-021-00812-w

**Published:** 2021-01-29

**Authors:** Jean-François Llitjos, Swann Bredin, Jean-Baptiste Lascarrou, Thibaud Soumagne, Mariana Cojocaru, Maxime Leclerc, Arnaud Lepetit, Albin Gouhier, Julien Charpentier, Gaël Piton, Matthieu Faron, Annabelle Stoclin, Frédéric Pène

**Affiliations:** 1 3i Department, Team Pulmonary and Systemic Immune Responses During Acute and Chronic Bacterial Infections, Institut Cochin, INSERM U1016, CNRS UMR8104, Université de Paris, Paris, France; 2 Medical Intensive Care Unit, Hôpital Cochin, Assistance Publique-Hôpitaux de Paris, APHP, Centre, Paris, France; 3 Intensive Care Unit, Hôpital Hôtel-Dieu, Nantes, France; 4 Intensive Care Unit, Hôpital Jean Minjoz Hospital, Besançon, France; 5 Surgical Intensive Care Unit, Hôpital Cochin, Assistance Publique-Hôpitaux de Paris, APHP, Centre, Paris, France; 6 Intensive Care Unit, Centre Hospitalier Mémorial France Etats-Unis, Saint-Lô, France; 7 Intensive Care Unit, Centre Hospitalier Intercommunal Alençon Mamers, Alençon, France; 8 Department of Biostatistics and Epidemiology, Inserm UNIT 1018 CESP Oncostat Team, Gustave Roussy Cancer Campus, Villejuif, France; 9 Intensive care unit, Gustave Roussy, Université Paris-Saclay, Villejuif, France

**Keywords:** COVID-19, Ventilator-acquired pneumonia, Immunosuppression, Septic shock

## Abstract

**Background:**

The aim of this study is to determine whether severe COVID-19 patients harbour a higher risk of ICU-acquired pneumonia.

**Methods:**

This retrospective multicentre cohort study comprised all consecutive patients admitted to seven ICUs for severe COVID-19 pneumonia during the first COVID-19 surge in France. Inclusion criteria were laboratory-confirmed SARS-CoV-2 infection and requirement for invasive mechanical ventilation for 48 h or more. Control groups were two historical cohorts of mechanically ventilated patients admitted to the ICU for bacterial or non-SARS-CoV-2 viral pneumonia. The outcome of interest was the development of ICU-acquired pneumonia. The determinants of ICU-acquired pneumonia were investigated in a multivariate competing risk analysis.

**Result:**

One hundred and seventy-six patients with severe SARS-CoV-2 pneumonia admitted to the ICU between March 1st and 30th June of 2020 were included into the study. Historical control groups comprised 435 patients with bacterial pneumonia and 48 ones with viral pneumonia. ICU-acquired pneumonia occurred in 52% of COVID-19 patients, whereas in 26% and 23% of patients with bacterial or viral pneumonia, respectively (*p* < 0.001). Times from initiation of mechanical ventilation to ICU-acquired pneumonia were similar across the three groups. In multivariate analysis, the risk of ICU-acquired pneumonia remained independently associated with underlying COVID-19 (SHR = 2.18; 95 CI 1.2–3.98, *p* = 0.011).

**Conclusion:**

COVID-19 appears an independent risk factor of ICU-acquired pneumonia in mechanically ventilated patients with pneumonia. Whether this is driven by immunomodulatory properties by the SARS-CoV-2 or this is related to particular processes of care remains to be investigated.

## Introduction

The coronavirus disease 2019 (COVID-19) pandemic caused by SARS-CoV-2 infection is responsible for severe pulmonary involvement frequently requiring intensive care unit (ICU) admission for advanced respiratory support [1]. It rapidly came out that this new pulmonary infection exhibited specific pathophysiological and clinical features, making it somewhat different from more classical bacterial and viral pneumonia. In the absence of efficient therapy, mechanical ventilation is the cornerstone of the COVID19 management but then exposes patients to ICU-acquired infections.

ICU-acquired pulmonary infections are major complications of invasive mechanical ventilation, responsible for respiratory deterioration, extra-pulmonary organ dysfunctions and prolongation of ventilation and of the length stay in the ICU. Hence, ICU-acquired pneumonia is a frequent complication in critically ill ventilated patients, with incidences ranging from 13.5 to 23%, and accounts for a major cause of morbidity and mortality in septic patients [2]. Prolonged mechanical ventilation represents the leading risk factor of ICU-acquired pneumonia. We also previously reported that septic shock patients with pneumonia exhibited the highest risk of ICU-acquired pneumonia, suggesting that a primary pulmonary insult may drive profound alterations in lung defence towards a secondary infectious insult [3]. As a matter of fact, sepsis-induced immunosuppression as assessed by quantitative and functional defects in circulating immune cells has been associated with increased susceptibility to secondary ICU-acquired infections (ICU-AI) [4]. Whether COVID-19 patients harbour different susceptibility towards secondary infections have not been investigated.

To address these questions, we investigated the incidence and the determinants of ICU-acquired pneumonia in mechanically ventilated COVID-19 patients, as compared to control patients with severe bacterial or viral pneumonia.

## Methods

### Study design and participants

We performed a retrospective study in seven ICUs within six French hospitals. The study gathered consecutive adult patients (aged ≥ 18 years old) admitted to the ICU with a PCR-confirmed SARS-CoV-2 pneumonia requiring mechanical ventilation. Only patients who received endotracheal intubation during the first two days following ICU admission and required mechanical ventilation for at least 48 h were included. COVID-19 patients were compared to mechanically ventilated patients with community-acquired bacterial or viral pneumonia, extracted from a retrospective 2008–2017 database of the medical ICU of Cochin hospital [3]. The Research Ethics Committee of the Institut Gustave Roussy approved the study and waived the need for patient’s consent. The study was registered at the French National Commission on Informatics and Liberty and at the French National Institute for Health Data. The ethics committee of the French Intensive Care Society had previously approved the constitution of the historical database (ref. CE SRLF, #16–30).

### Data collection

Demographic, clinical, laboratory, treatment and organ support at baseline, and outcome data were collected from electronic medical records using a standardized data collection form. Given the protracted mechanical ventilation that were observed in COVID-19 patients at the beginning of the outbreak [5], the observation period was extended to day 45.

### Definitions

Laboratory confirmation of COVID-19 was based upon SARS-CoV-2 detection by real-time RT-PCR from nasal swabs or lower respiratory tract secretions. Obesity was defined as a body mass index superior to 30 kg/m^2^. Patients were considered immunocompromised if one or more of the following conditions were observed: patients with solid tumours with chemotherapy in the last 3 months or a progressive metastatic disease, hematologic malignancies, solid organ transplantation, HIV infection with or without AIDS, treatment with corticosteroids (> 3 months at any dosage or ≥ 1 mg/kg prednisone equivalent per day for > 7 day), or treatment with other immunosuppressive drugs. Severity at admission was assessed by the Simplified Acute Physiology Score 2 and the Sequential Organ Failure Assessment (SOFA) scores. Acute respiratory distress syndrome (ARDS) was diagnosed according to the Berlin definition [6].

ICU-acquired pneumonia was defined as new onset of probable or definite infection not present at the time of ICU admission and that developed after the first 48 h from ICU admission. Only the first episode of ICU-acquired pneumonia was considered for the present analysis. ICU-acquired pneumonia diagnosis was based on a Clinical Pulmonary Infectious Score > 6 [7]. Patients with clinically suspected ICU-acquired pneumonia were subjected to a tracheobronchial aspirate or broncho-alveolar lavage with direct Gram staining and semi-quantitative culture [7]. Invasive fungal infections were diagnosed according to current guidelines [8]. An independent physician (JFL) retrospectively assessed the diagnostic accuracy of all episodes of ICU-acquired pneumonia.

### Patient management

Management of sepsis and septic shock was in accordance with the guidelines of the Surviving Sepsis Campaign [9]. Antimicrobial treatments were administered intravenously depending on the clinical suspicion of infection and known colonization with antibiotic-resistant bacteria and deescalated to narrower spectrum after identification of the responsible pathogen. Management of ARDS in the COVID-19 cohort followed the French Intensive Care Society guidelines [10]. Strategies to prevent ICU-acquired pneumonia were implemented according to guidelines and included the use of weaning protocols, semi-recumbent position, enteral route feeding and physiotherapy [7]. End-of-life decisions to withhold or withdraw life support were taken independently at each centre and palliative care was then appropriately delivered in the ICU.

### Statistical analysis

Continuous variables were expressed as median (interquartile range) and categorical variables as numbers (percentages) and were compared by the Kruskal–Wallis’ test, the Pearson’s Chi-square test or the Fisher’s exact test as appropriate. The independent predictors of ICU death were investigated through a multivariate Fine–Gray model analysis to fit cumulative incidence curves. The model included variables that reached p value less than 0.20 in univariate analysis.

Determinants of ICU-acquired pneumonia were analysed through a competing risk framework, with death in ICU and extubation for more than 48 h as competing events. Independent determinants of ICU-acquired pneumonia were investigated in a multivariate analysis using a Fine–Gray model to fit cumulative incidence curves. We also investigated the determinants of ICU-acquired pneumonia using a cause-specific Cox model. The model included variables that reached p values of less than 0.20 in univariate analysis and proportional hazard assumption was checked using graphical diagnostics based on the scaled Schoenfeld residuals. All analyses were carried out using R 3.3.3 (R foundation for Statistical Computing Vienna, Austria).

## Results

### COVID-19 and non-COVID-19 cohorts

Between March 1st and 30th June of 2020, 176 patients were admitted in ICU for severe SARS-CoV-2 pneumonia requiring invasive mechanical ventilation for more than 48 h. Their main characteristics are summarized in Table 1. Arterial hypertension and obesity were the prominent comorbid conditions. Time from symptoms onset to ICU admission was 7.5 (4.25–10) days. About one-third of COVID-19 patients (*n* = 66, 37%) received specific antiviral treatments, including hydroxychloroquine and azithromycin combination in 39 patients, lopinavir in 18 patients and remdesivir in 8 patients. Twelve COVID-19 patients (6%) were treated with steroids (dexamethasone) and one patient received tocilizumab. Bacterial co-infection was documented in 21 (12%) patients. Nearly all patients fulfilled the criteria for ARDS (*n* = 170, 96%) and most of them underwent prone positioning (*n* = 130, 74%) with a median number of 3 (1–7) sessions. Rescue veno-venous extracorporeal membrane oxygenation (ECMO) was used in 10 patients. The overall ICU mortality rate was 31% (*n* = 55) and the median duration of mechanical ventilation was 17 days (25th–75th IQR: 10–28). Half of patients (49%) with bacterial pneumonia received stress-dose hydrocortisone for acute circulatory failure.

**Table 1:** Characteristics and outcome of patients with COVID-19, bacterial pneumonia or viral pneumonia

Variables	COVID-19 (*n* = 176)	Bacterial pneumonia (*n* = 435)	Viral pneumonia (*n* = 48)	*p*
Age, years	63 (55–73)	66 (56–79)	72 (42–75)	0.002
Male gender	134 (76)	296 (68)	25 (52)	< 0.001
Body mass index, kg/m^2^	28.2 (26–32.3)	23.4 (20.7–26.7)	23 (19.2–28.9)	< 0.001
Comorbid conditions				
Immunosuppression	26 (15)	145 (33)	13 (27)	< 0.001
Cirrhosis	2 (1)	41 (9)	1 (2)	< 0.001
Diabetes mellitus	45 (26)	89 (21)	7 (14)	0.181
COPD	17 (10)	103 (24)	9 (19)	< 0.001
Chronic renal failure	13 (7)	49 (11)	7 (15)	0.22
Cancer (ongoing or < 5 years)	16 (9)	120 (28)	8 (17)	< 0.001
Severity on ICU admission				
SAPS2, points	43 (30–56)	73 (55–88)	47 (39–71)	< 0.001
SOFA, points	6 (4–9)	9 (6– 2)	7 (2–13)	< 0.001
Biological findings				
WBC count, per mm^3^	8.4 (6.9–12.4)	12 (6.2–17.6)	8.8 (3.4–11.7)	< 0.001
Lactate, mmol/L	1.5 (1.2–1.9)	1.7 (1–3.8)	1.4 (0.4–3.1)	0.02
Characteristics of pneumonia				
Clinical presentation				
Septic shock (Sepsis-3)	28 (16)	191 (44)	15 (31)	< 0.001
ARDS	170 (96)	62 (14)	17 (35)	< 0.001
Microbiological documentation	21 (12)	286 (66)	11 (24)	< 0.001
Gram-negative bacteria	11 (7)	148 (34)	5 (10)	
Gram-positive cocci	10 (6)	126 (30)	6 (13)	
SARS-CoV-2	176 (100)	0	0	
I nfluenza virus	0	0	46 (96)	
Miscellaneous	0	12 (3)*	2 (4)**	
ICU management within the first 48 h				
Antimicrobial agents	161 (92)	435 (100)	45 (94)	< 0.001
Vasopressors	371 (85)	147 (83)	37 (71)	0.32
Prone positioning	130 (74)	41 (9)	9 (19)	< 0.001
Number of sessions	3 (2–5)	2 (1,5–2,5)	1 (1–2)	< 0.001
Anaesthesia ventilator	12 (7)	0 (0)	0 (0)	< 0.001
Extracorporeal membrane oxygenation	10 (6)	14 (3)	5 (10)	0.04
Renal replacement therapy	51 (29)	44 (10)	9 (19)	< 0.001
Blood transfusions	67 (38)	223 (51)	18 (37)	0.01
Corticosteroids	12 (6)	213 (49)	0 (0)	< 0.001
Outcomes				
Total duration of mechanical ventilation, days	17 (10–28)	8 (5–16)	7 (4–13)	< 0.001
ICU length of stay, days	20 (12–30)	10 (6–19))	9 (6–16)	< 0.001
ICU mortality	55 (31)	143 (33)	12 (25)	< 0.001

ICU, intensive care unit, ICU-AI, intensive care unit-acquired infections, COPD, chronic obstructive pulmonary disease, SAPS2, Simplified Acute Physiology Score 2, SOFA, Sequential Organ Failure Assessment score, WBC, white blood cell. Variables are expressed as median (interquartile range) or number (percentage) as appropriate

^*^ Including fungi (*n* = 10) and mycobacteria (*n* = 2), **Including metapneumovirus (*n* = 1) and cytomegalovirus (*n* = 1)

Control groups were patients with severe bacterial (*n* = 435) or viral (*n* = 48) pneumonia. Their characteristics are displayed in Table 1, and compared with those of COVID-19 patients. All cases of viral pneumonia were related to influenza virus, except two episodes related to metapneumovirus and cytomegalovirus. Bacterial co-infection was documented in 11 (24%) patients with viral pneumonia. The overall ICU mortality was 33% in patients with bacterial pneumonia and 25% in viral pneumonia.

COVID-19 and non-COVID-19 patients markedly differ in a number of underlying characteristics and clinical presentation of acute condition (Table 1). Although COVID-19 patients exhibited lower admission severity scores, they more often fulfilled the diagnostic criteria for ARDS. COVID-19 finally exhibited increased duration under mechanical ventilation (median 17 days vs. 8 days in bacterial pneumonia and 7 days in viral pneumonia, *p* < 0.001) and eventually increased length of stay in the ICU (median 20 days vs. 10 days in bacterial pneumonia and 9 days in viral pneumonia, *p* < 0.001). In-ICU mortality rates of COVID-19 and bacterial pneumonia were similar (31% and 33%, respectively), both slightly higher than that of viral pneumonia (25%). However, the mortality rates did not differ across even after adjustment with other confounders (Additional file 1: Table S1).

### ICU-acquired infections in COVID-19 and non-COVID-19 patients

**Fig. 1:**
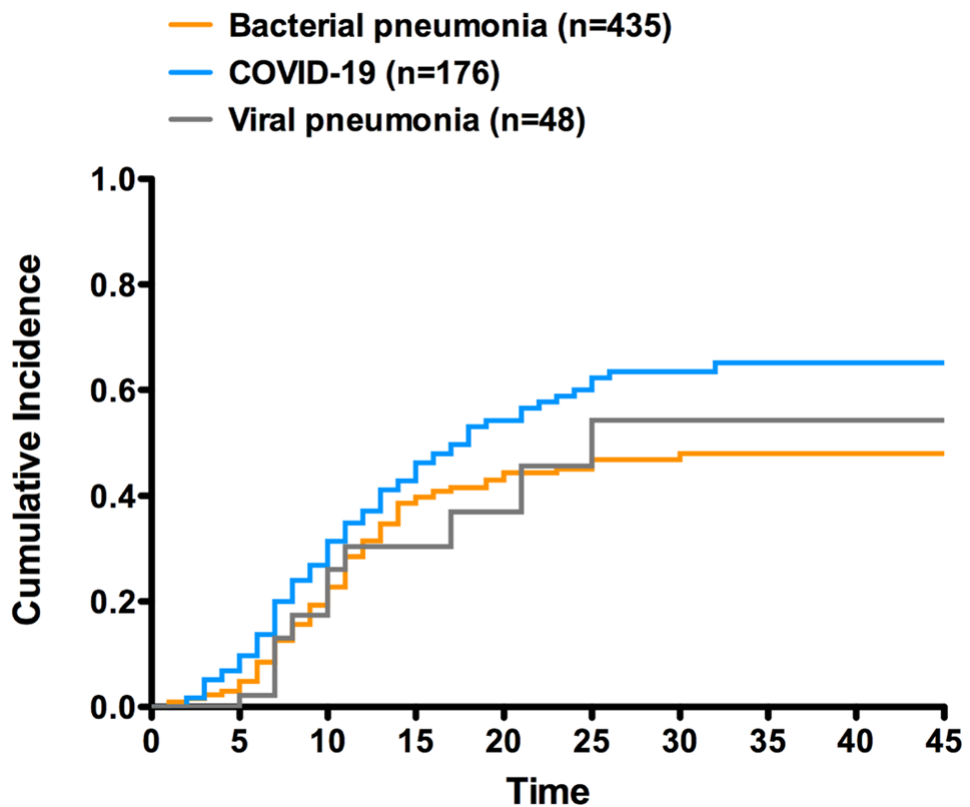
Cumulative incidence of ventilator-associated pneumonia among patients

The frequency of ICU-acquired pneumonia was 52% in COVID-19 patients, with a median time from intubation to diagnosis of 9 (6–14) days (Fig. 1). ICU-acquired pneumonia was responsible for deterioration to septic shock in half of cases. Patients with bacterial pneumonia and viral pneumonia exhibited lower ICU-acquired pneumonia frequencies of 26% and 23%, respectively (Table 2). Times from intubation to ICU-acquired pneumonia were 9 (6–12) days in bacterial pneumonia and 7 (6.5–14) days in viral pneumonia, thereby similar to that of COVID-19 patients. The distribution of causing pathogens is precised in Table 2. In multivariate analysis taking into account mechanical ventilation as a time-dependent variable, the independent determinants of ICU-acquired pneumonia were COVID-19 pneumonia (SHR = 2.18; 95% CI 1.2–3.98, *p* = 0.011), male gender (SHR = 1.55; 95% CI 1.14–2.12, *p* = 0.005), ARDS (SHR = 1.84; 95% CI 1.25–2.72, p = 0.002) and duration of mechanical ventilation (SHR = 1.027, 95% CI 1.01–1,04, *p *≤ 0.001) (Table 3). Of note, because control cohorts were obtained from one centre, this variable was considered in our statistical analysis and was not associated with ICU-acquired pneumonia.

**Table 2:** Intensive care unit-acquired pneumonia (ICU-AP) in patients

ICU-acquired pneumonia	COVID-19 (*n* = 92)	Bacterial pneumonia (*n* = 113)	Viral pneumonia (*n* = 11)	*p*
Frequency	92 (52)	113 (26)	11 (23)	< 0.001
Time from intubation to first ICU-AP, days	9 (6–14)	9 (6–12)	7 (6.5–14)	0.70
Deterioration to septic shock	45 (49)	72 (64)	5 (45)	0.01
Microbiological documentation				
Enterobacteriaceae	50	27	3	< 0.001
Non-fermenting GNB	20	114	7	
Gram-positive cocci	28	3	1	
Polymicrobial	24 (16)	19 (17)	2 (4)	
Fungi	1	2	0	
Subsequent episodes of ICU-AP				
Second	37 (21)	53 (12)	3 (6)	
Third	15 (8)	37 (8)	3 (6)	
Fourth	2 (1)	12 (3)	0	

ICU, intensive care unit, ICU-AP, intensive care unit-acquired pneumonia. Variables are expressed as median (interquartile range) or number (percentage) as appropriate

**Table 3:** Determinants associated with ICU-acquired pneumonia using sub-distribution hazard ratio in univariate and multivariate analysis

Variables group	Univariate			Multivariate		
	SHR	95% CI	*p*	SHR	95% CI	*p*
Group						
Bacterial pneumonia	Ref	Ref	Ref	Ref	Ref	Ref
COVID-19	1.52	1.17–1.98	0.001	2.18	1.2–3.98	0.011
Viral pneumonia	0.8	0.46–1.42	0.46	0.76	0.43–1.36	0.35
Male gender	1.67	1.23–2.27	0.001	1.55	1.14–2.12	0.005
Obesity	1.25	0.96–1.63	0.094			
Immunosuppression	0.68	0.5–0.93	0.02			
Admission SAPS2, per point	0.99	0.99–1	0.06			
Admission SOFA, per point	0.97	0.95–1.01	0.15			
COPD	0.65	0.45–0.97	0.032			
Cancer	0.73	0.52–1.04	0.081			
Prone positioning	1.03	1–1.07	0.075			
Vasopressors	1.42	0.97–2.08	0.073			
Blood transfusion	1.21	0.93–1.57	0.15			
Conventional respiratory device	0.39	0.21–0.73	0.003			
Antimicrobial treatment	0.46	0.24–0.9	0.023			
ARDS	1.61	1.39–2.37	< 0.001	1.84	1.25–2.72	0.002
Duration of MV prior to VAP, days	1.02	1.02–1.04	< 0.001	1.027	1.01–1.04	< 0.001

SAPS2, Simplified Acute Physiology Score 2; COPD, chronic obstructive pulmonary disease; ARDS, acute respiratory distress syndrome; MV, mechanical ventilation. All covariates were analysed at baseline except for mechanical ventilation that was evaluated as time-dependent covariate

## Discussion

COVID-19 pandemic has shed light on the risk of shortage in ICU beds related to the surge of ICU admissions, but also to prolonged bed occupancy by patients with sustained respiratory failure. Protracted mechanical ventilation in SARS-CoV-2 pneumonia can be primarily ascribed to the severity of the lung injury, but also to the development of secondary complications. In this study, we addressed the specific risk of ICU-acquired pneumonia in the course of severe. When compared to recent cohorts of patients with non-COVID-19 pneumonia, patients with severe SARS-CoV-2 pulmonary infection exhibited a prominent risk of ICU-acquired pneumonia.

Other studies have already reported various frequency rates of ICU-acquired pneumonia in mechanically ventilated COVID-19 patients, with estimated incidence ranging from 16 to 31% in two early reports from China [11, 12]. Since then, several studies confirmed this impression [13–16]. Importantly, an accurate estimation for the cumulative risk of ICU-acquired pneumonia in ventilated patient requires handling two main competing events that are extubation and death to avoid misinterpretation of risks. The high rate of ICU-acquired pneumonia in COVID-19 patients may result from a particular susceptibility to pulmonary superinfections. The question is whether COVID-19 stands as risk factor of ICU-acquired infections on its own, or if this association is rather related to confounding factors such as exposure to invasive devices or significant changes in care practice. Such a retrospective study can hardly provide a definite answer between causality and association, despite investigating the potential confounders and entering them into multivariate models. Since ICU-acquired pneumonia is strongly associated with the duration under invasive mechanical ventilation, we treated mechanical ventilation as a time-dependent covariate in multivariate analysis [17]. Nonetheless, it is noteworthy that times from intubation to the first episode of ICU-acquired pneumonia were similar across the three groups. Of note, nearly all patients from the three groups had received antibiotics in the early days in the ICU. The increased risk of ICU-acquired pneumonia may also be related to increased workload that may have prevented strict and thorough implementation of preventive bundles.

COVID-19 has emerged as a particular infection with a characteristic two-step course in a significant proportion of patients. Whereas the primary symptoms are associated with viral shedding, secondary respiratory deterioration is associated with potent systemic acute inflammatory response. The pathophysiology of COVID-19 lung involvement encompasses endothelial and epithelial alterations as well as pulmonary embolism and microvascular thrombosis. Besides, secondary infectious insults sustain acute lung injury and likely contribute to prolonged mechanical ventilation. The particular susceptibility of post-COVID critically ill patients to ICU-acquired pneumonia suggests defective anti-infective immune responses against bacterial superinfections reminiscent of those observed in post-septic patients [18]. Sepsis-induced immunosuppressive response is related to various quantitative and functional alterations in most immune cells [4]. Whether such immune dysfunctions may also account for increased susceptibility to secondary infections in COVID-19 patients remains to be investigated.

Interventional studies for severe COVID-19 have so far attempted dampening the primary pro-inflammatory cytokine response by anti-inflammatory compounds, most especially to prevent the respiratory deterioration of patients with mild pneumonia. There is no signal so far that such early immunomodulatory therapeutics of COVID-19 may increase the risk of secondary infections. However, the particular susceptibility of COVID-19 patients to secondary bacterial infections raises the question of immunostimulant strategies later on in the ICU. For instance, drugs known to restore monocyte functions and HLA-DR and CD14 expressions such as interferon gamma (IFNγ) or GM-CSF, or drugs to restore lymphocyte activation such as IL-7 or thymosin-α may represent attractive therapeutic options in this setting [19].

This study has several limitations. Although improving the external validity, the multicentre design also harbours the risk of inconsistent care and diagnostic procedures. The diagnosis of ICU-acquired pneumonia was let at the discretion of the physician in charge, with the help of a validated score, and was reviewed by an independent investigator to ensure appropriate and consistent diagnosis. Data from severe COVID-19 patients were obtained from seven different ICUs, whereas controls with bacterial and viral pneumonia were obtained from one single centre. However, frequencies of secondary pneumonia in COVID-19 patients were consistent across centres, and the centre effect was taken into account in the multivariate models. Most importantly, such a retrospective study is limited to establish a definite causality inference, although we aimed at taking into account major determinants of ICU-acquired pneumonia in the multivariate model.

## Conclusion

We identified SARS-CoV-2 infection as an independent risk factor of ICU-acquired pneumonia among mechanically ventilated patients with common bacterial and viral causes of pneumonia. This justifies a thorough reinforcement of preventive measures in these high-risk patients. In the light of immunomodulatory approaches to treat COVID-19, our results raise the question of immunostimulant therapies to fight bacterial superinfections in critically ill COVID-19 patients.

## Supplementary Information

**Additional file 1: Table S1. **Determinants of ICU mortality using sub-distribution hazard ratio (SHR) in univariate and multivariate analysis.

## Data Availability

Data and material are available under request.

## References

[1] Huang C, Wang Y, Li X, Ren L, Zhao J, Hu Y (2020). Clinical features of patients infected with 2019 novel coronavirus in Wuhan, China. Lancet.

[2] Melsen WG, Rovers MM, Groenwold RHH, Bergmans DCJJ, Camus C, Bauer TT (2013). Attributable mortality of ventilator-associated pneumonia: a meta-analysis of individual patient data from randomised prevention studies. Lancet Infect Dis.

[3] Llitjos J-F, Gassama A, Charpentier J, Lambert J, de Roquetaillade C, Cariou A (2019). Pulmonary infections prime the development of subsequent ICU-acquired pneumonia in septic shock. Ann Intensive Care.

[4] Hotchkiss RS, Monneret G, Payen D (2013). Immunosuppression in sepsis: a novel understanding of the disorder and a new therapeutic approach. Lancet Infect Dis.

[5] Bhatraju PK, Ghassemieh BJ, Nichols M, Kim R, Jerome KR, Nalla AK (2020). Covid-19 in critically ill patients in the Seattle Region—case series. N Engl J Med.

[6] Acute Respiratory Distress Syndrome: the Berlin Definition. JAMA. 2012 (cited 2020 May 29);307(23). http://jama.jamanetwork.com/article.aspx?doi=10.1001/jama.2012.5669 10.1001/jama.2012.5669 22797452

[7] Leone M, Bouadma L, Bouhemad B, Brissaud O, Dauger S, Gibot S, et al. Pneumonies associées aux soins de réanimation* RFE commune SFAR–SRLF. Mootien J, Bretonnière C, editors. Médecine Intensive Réanimation. 2019;28(3):261–81.

[8] De Pauw B, Walsh TJ, Donnelly JP, Stevens DA, Edwards JE, Calandra T (2008). Revised definitions of invasive fungal disease from the European Organization for Research and Treatment of Cancer/Invasive Fungal Infections Cooperative Group and the National Institute of Allergy and Infectious Diseases Mycoses Study Group (EORTC/MSG) Consensus Group. Clin Infect Dis Off Publ Infect Dis Soc Am.

[9] Rhodes A, Evans LE, Alhazzani W, Levy MM, Antonelli M, Ferrer R (2017). Surviving sepsis campaign: International Guidelines for Management of Sepsis and Septic Shock: 2016. Crit Care Med.

[10] Papazian L, Aubron C, Brochard L, Chiche J-D, Combes A, Dreyfuss D (2019). Formal guidelines: management of acute respiratory distress syndrome. Ann Intensive Care.

[11] Zhou F, Yu T, Du R, Fan G, Liu Y, Liu Z (2020). Clinical course and risk factors for mortality of adult inpatients with COVID-19 in Wuhan, China: a retrospective cohort study. Lancet.

[12] Ruan Q, Yang K, Wang W, Jiang L, Song J (2020). Clinical predictors of mortality due to COVID-19 based on an analysis of data of 150 patients from Wuhan, China. Intensive Care Med.

[13] on behalf of the coVAPid study Group, Rouzé A, Martin-Loeches I, Povoa P, Makris D, Artigas A, et al. Relationship between SARS-CoV-2 infection and the incidence of ventilator-associated lower respiratory tract infections: a European multicenter cohort study. Intensive Care Med. 2021(cited 2021 Jan 8). http://link.springer.com/10.1007/s00134-020-06323-9 10.1007/s00134-020-06323-9 PMC7778569 33388794

[14] Razazi K, Arrestier R, Haudebourg AF, Benelli B, Carteaux G, Decousser J (2020). Risks of ventilator-associated pneumonia and invasive pulmonary aspergillosis in patients with viral acute respiratory distress syndrome related or not to coronavirus 19 disease. Crit Care..

[15] Luyt C-E, Sahnoun T, Gautier M, Vidal P, Burrel S, de Chambrun PM (2020). Ventilator-associated pneumonia in patients with SARS-CoV-2-associated acute respiratory distress syndrome requiring ECMO: a retrospective cohort study. Ann Intensive Care..

[16] COVID-ICU Group on behalf of the REVA Network and the COVID-ICU Investigators. Clinical characteristics and day-90 outcomes of 4244 critically ill adults with COVID-19: a prospective cohort study. Intensive Care Med. 2020. 10.1007/s00134-020-06294-x PMC7674575 33211135

[17] van Vught LA, Klein Klouwenberg PMC, Spitoni C, Scicluna BP, Wiewel MA, Horn J (2016). Incidence, risk factors, and attributable mortality of secondary infections in the intensive care unit after admission for sepsis. JAMA.

[18] Boomer JS, To K, Chang KC, Takasu O, Osborne DF, Walton AH (2011). Immunosuppression in patients who die of sepsis and multiple organ failure. JAMA.

[19] Payen D, Faivre V, Miatello J, Leentjens J, Brumpt C, Tissières P (2019). Multicentric experience with interferon gamma therapy in sepsis induced immunosuppression. A case series. BMC Infect Dis..

