# Supplementary material for: Increased susceptibility to intensive care unit-acquired pneumonia in severe COVID-19 patients: a multicentre retrospective cohort study
Source: Ann Intensive Care. 2021 Jan 29;11:20. doi: 10.1186/s13613-021-00812-w (PMC7844782; doi:10.1186/s13613-021-00812-w)
Supplement: Supplementary file 1 — Additional file 1: Table S1. Determinants of ICU mortality using sub-distribution hazard ratio (SHR) in univariate and multivariate analysis. [file 13613_2021_812_MOESM1_ESM.docx]

| **Variables** | **Univariate** | | |  | **Multivariate** | | |
| --- | --- | --- | --- | --- | --- | --- | --- |
|  | **SHR** | **95% CI** | **p** |  | **SHR** | **95% CI** | **p** |
| **Group** |  |  |  |  |  |  |  |
| **Bacterial pneumonia** | **ref** | **ref** | **ref** |  |  |  |  |
| **COVID-19** | **0.91** | **0.68 - 1.24** | **0.58** |  |  |  |  |
| **Viral pneumonia** | **0.76** | **0.42 - 1.38** | **0.37** |  |  |  |  |
| **Age, per year** | **1.03** | **1.02 - 1.04** | **<0.001** |  | **1.03** | **1.02 - 1.04** | **<0.001** |
| **Admission SAPS2, per point** | **1.01** | **1.01 - 1.02** | **<0.001** |  |  |  |  |
| **Admission SOFA, per point** | **1.03** | **1 - 1.07** | **0.004** |  |  |  |  |
| **Immunosuppression** | **1.32** | **1 - 1.77** | **0.05** |  |  |  |  |
| **Chronic renal failure** | **1.44** | **0.97 - 2.16** | **0.071** |  |  |  |  |
| **Cancer** | **1.45** | **1.07 - 1.96** | **0.016** |  |  |  |  |
| **Vasopressors** | **0.74** | **0.53 - 1.04** | **0.086** |  | **0.57** | **0.39 - 0.86** | **0.007** |
| **Admission lactate level, per mmol/L** | **1.07** | **1.01 - 1.15** | **0.027** |  | **1.076** | **1.01 - 1.15** | **0.02** |
| **ARDS** | **1.26** | **0.97 - 1.66** | **0.042** |  | **2.2** | **1.56 - 3.11** | **<0.001** |
| **Transfusion** | **1.63** | **1.24 - 2.16** | **<0.001** |  | **1.97** | **1.41 - 2.75** | **<0.001** |
| **Duration of MV, days** | **0.99** | **0.98 - 1** | **0.07** |  | **0.97** | **0.96 - 0.99** | **<0.001** |

**Table S1: Determinants of ICU mortality using sub-distribution hazard ratio (SHR) in univariate and multivariate analysis.**

SAPS II: Simplified Acute Physiology Score, SOFA: Sepsis-related Organ Failure Assessment, ARDS: acute respiratory distress syndrome, MV: mechanical ventilation.
